# The Medical Age

**Published:** 1883-11

**Authors:** 


### THE MEDICAL AGE.

The editor of this spicy journal sends us an amendment to our proposition anent the medical colleges of Louisville, Ky. He amended by putting the number to be retired or shot out of the professional muddle, at a third instead of every tenth man. We accept, and propose that the execution be handed over to the Illinois Board of Health.

